# Supplementary material for: Spatial and temporal expansion of global wildland fire activity in response to climate change
Source: Nat Commun. 2022 Mar 8;13:1208. doi: 10.1038/s41467-022-28835-2 (PMC8904637; doi:10.1038/s41467-022-28835-2)
Supplement: Supplementary file 1 — Supplementary Information [file 41467_2022_28835_MOESM1_ESM.pdf]

Supplementary Information for

Spatial and temporal expansion of global wildland fire activity in response to climate change

Martín Senande-Rivera[1*], Damián Insua-Costa[1] and Gonzalo Miguez-Macho[1].

[1]Nonlinear Physics Group, Faculty of Physics, Universidade de Santiago de Compostela, Galicia, Spain.

[*]Correspondence to: martin.senande.rivera@usc.es

Burned area data

The fire season length (FSL) is defined as the minimum number of months that contain more than 80% of the annual burned area. A similar definition, considering only consecutive months, is widely used[1-3]. We allow non-consecutive months in our definition to accurately identify bi-modal fire seasons. To calculate the FSL, we employ monthly means of burned area computed over years with total BA≥100 ha. We start from the month with highest value and progress to lower BA months until 80% or more of the total area is covered. Supplementary Fig. 1a shows the FSL obtained from GFED4 burned area data.

The GFED4 dataset[4] contains information about the land cover distribution within the burned area. Supplementary Fig. 1b shows the percentage of the mean annual burned area (BA) associated to croplands (one of the land cover categories of the GFED4 data). Fires on agricultural land can correspond to human-controlled agricultural practices, with minimal relationship to weather or climate conditions.

Supplementary Fig. 2a shows the mean annual burned area. There are regions (e.g. boreal regions) with a high difference between mean and maximum annual burned area for the period (Fig. 1a of the main text), implying that there is large variability, with years when fire activity is particularly high among many other years with low burned area. Conversely, the more similar mean and maximum values in the tropical regions suggest that there the annual burned area is regularly high for most of the years. Similar conclusions can be inferred from Fig, S2b for the number of years per decade with an annual burned area higher than 100ha. The frequency of fires is much higher in the Tropics than anywhere else.

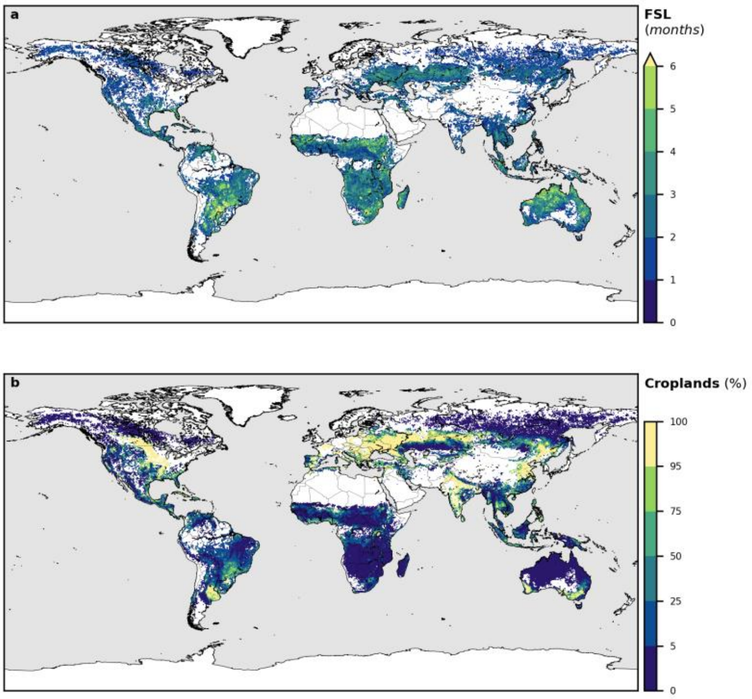

Supplementary Figure 1. a Fire season length. **b** Percentage of mean annual burned area (BA) corresponding to cropland land cover from GFED4 data[4].

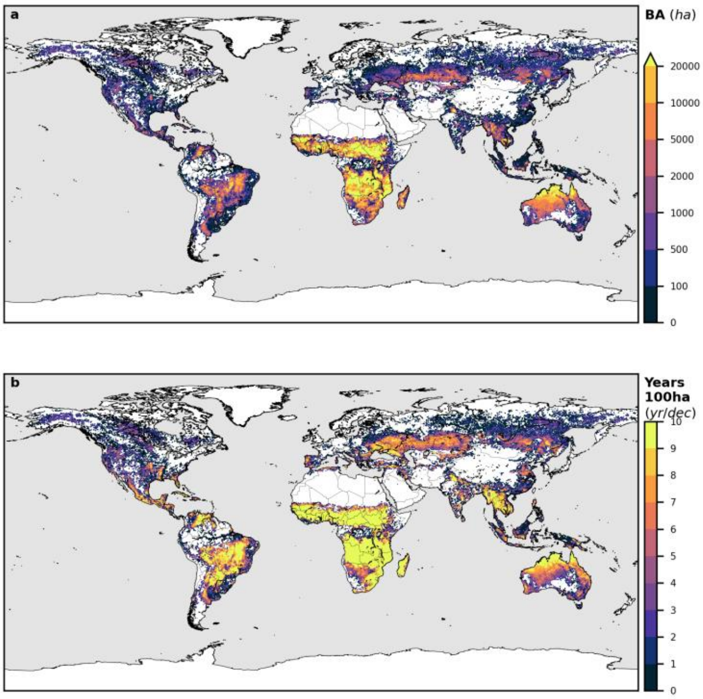

Supplementary Figure 2. a Mean annual burned area. **b** Number of years with an annual burned area >100ha per decade.

Köppen-Geiger classification

We sort out global grid points with burned area data into 5 different groups according to their main Köppen-Geiger climate class[5]: tropical (A), arid (B), temperate (C), cold (D) and polar (E). We follow the classification criteria used by Beck et al.[6] and Peel et al.[7], but with a new threshold between temperate (C) and cold (D) climates. Since the definition of the Köppen-Geiger climate classification several authors proposed changes, and most of these proposals were aimed at improving the C-D boundary[8], such as redefining the coolest-month isotherm at 0°C[6,7,9] or -3°C[10]. We modified the C-D boundary to associate the cold climate (D) to the boreal forest (taiga), so we set the threshold between temperate and cold climates at 2°C of mean annual temperature (MAT). This threshold is consistent with Whittaker[11] and Woodward et al.[12], who determine the temperate-boreal forest transition to occur below 5°C of mean annual temperature. Supplementary Fig. 3 shows the Köppen-Geiger world classification with this modification, computed from WFDE5 climate data[13].

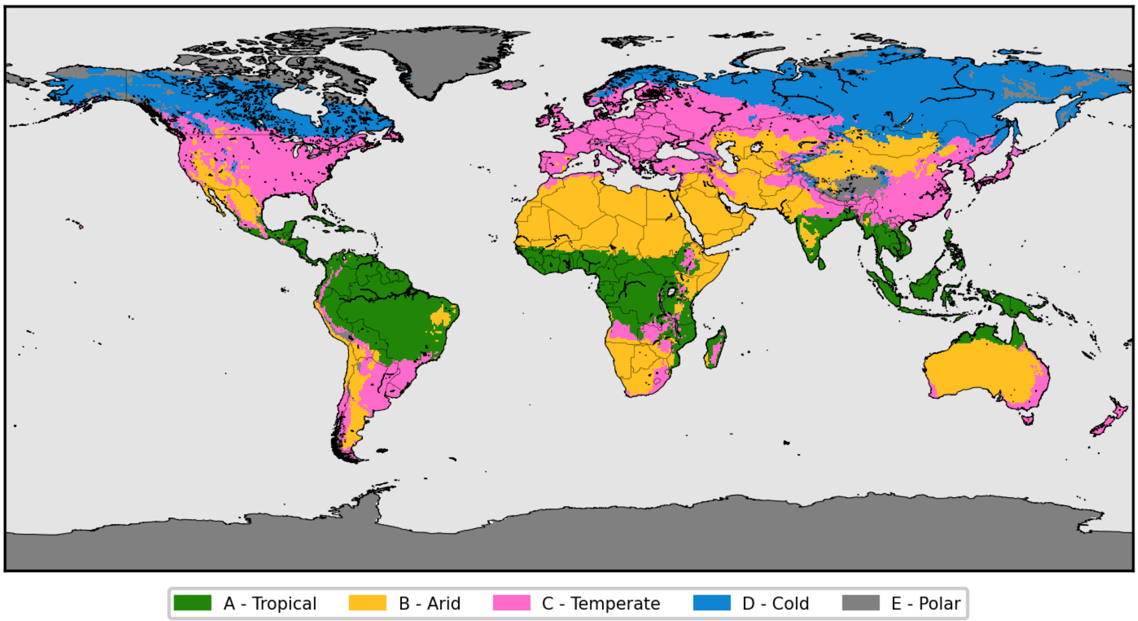

Supplementary Figure 3. World general climate classification. Based on the Köppen-Geiger classification but modifying the C-D category threshold to delimit the boreal forest ecoregion.

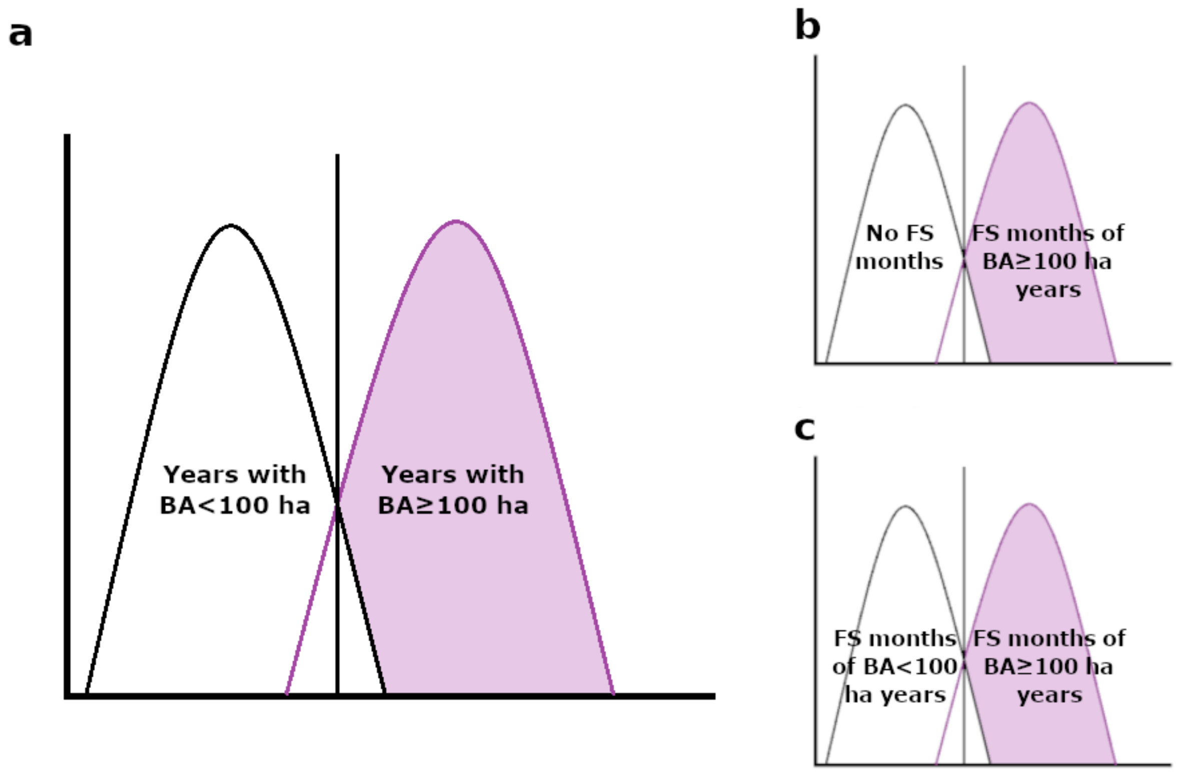

Supplementary Figure 4. Statistical distributions scheme. a Annual variables (Pa, Ta, Pmin and Tmax) are split into values for BA≥100 ha years and values for BA<100 ha years, all grid cells within the same main climate class are considered. Monthly variables (Pm and Tm) are used for two different comparisons: **b** seasonal and **c** interannual. The monthly seasonal distribution (**b**) compares the FS months of the BA≥100 ha years against the months that are not part of a FS. For cells with no fire incidence, there is no FS, and all months are included. All grid cells within the same main climate class are considered. The interannual distribution (**c**) compares the FS months of years with BA≥100 ha against the FS months of years with BA<100 ha for cells with some fire incidence within the same main climate class. The thresholds are selected automatically for each distribution at the value that maximizes the area enclosed between the density function of the points with high fire activity and the density function of the points with low or no fire activity.

Threshold selection for each climate

Tropical dry season fire category

Fire activity in the Tropics is confined to relatively dry months, in which the mean monthly precipitation (Pm) is less than 90 mm (Supplementary Fig. 6a). This variable is the one presenting more differences between the fire-prone and the non-fire probability density functions (area difference of 0.55, see Supplementary Fig. 6a). As many as 83.7% of the points with high fire activity, but only 28.4% of the non-fire points, are to the left of the threshold, accounting for 96.8% of the tropical burned area. The statistical distribution of Pmin (Supplementary Fig. 5c), with an area difference of 0.30, indicates that under tropical climates fire activity occurs in places where at least one month is extremely dry (< 6 mm). This implies that places where the dry season consists of months with mean monthly precipitations ranging between 6 mm and 90 mm, will not be classified as fire-prone. A stronger precipitation seasonality is needed, where at least one month has a mean precipitation under 6 mm. If this Pmin threshold is satisfied, the number of months with Pm < 90 mm is defined as the potential fire season (PFS) under tropical climates.

The Pa statistical distribution (Supplementary Fig. 5a) shows that fire-prone areas have relatively lower annual precipitations than the non-fire-prone regions; however, since this is likely due to the aforementioned rainfall seasonality, we find that we do not need to use an upper Pa limit to characterize the fire-prone regions in the Tropics. Neither we need to employ temperature variables in the fire-climate classification, as they do not show any distinct behaviour in the fire season (Supplementary Fig. 5b,d and Supplementary Fig. 6b,d). The strong link between fires in the Tropics and rainfall seasonality has been widely studied[14-16]. The location of the Inter-Tropical Convergence Zone (ITCZ) generally determines precipitation patterns in the Tropics, and consequently fire activity[16].

Fires in the Tropics occur with a very high annual frequency (Supplementary Fig. 2b). Lying close the equator, South-eastern maritime Asia (Indonesia, Malaysia and Papua New Guinea) is a region that is regularly not fire-prone, except when fire activity is favoured by the appearance of marked dry seasons associated with El Niño events, as suggested by the coincidence of low precipitation periods and high fire activity during the El Niño episodes of 1997/1998, 2002/2003, 2009/2010 and 2015/2016[4,17,18] (Supplementary Fig. 7). Fig. 2b of the main text shows this region classified as Tr-ds-i, indicating that climate conditions are prone

to fire activity with a frequency of 1 to 3 years per decade (a frequency interval consistent with El Niño episodes).

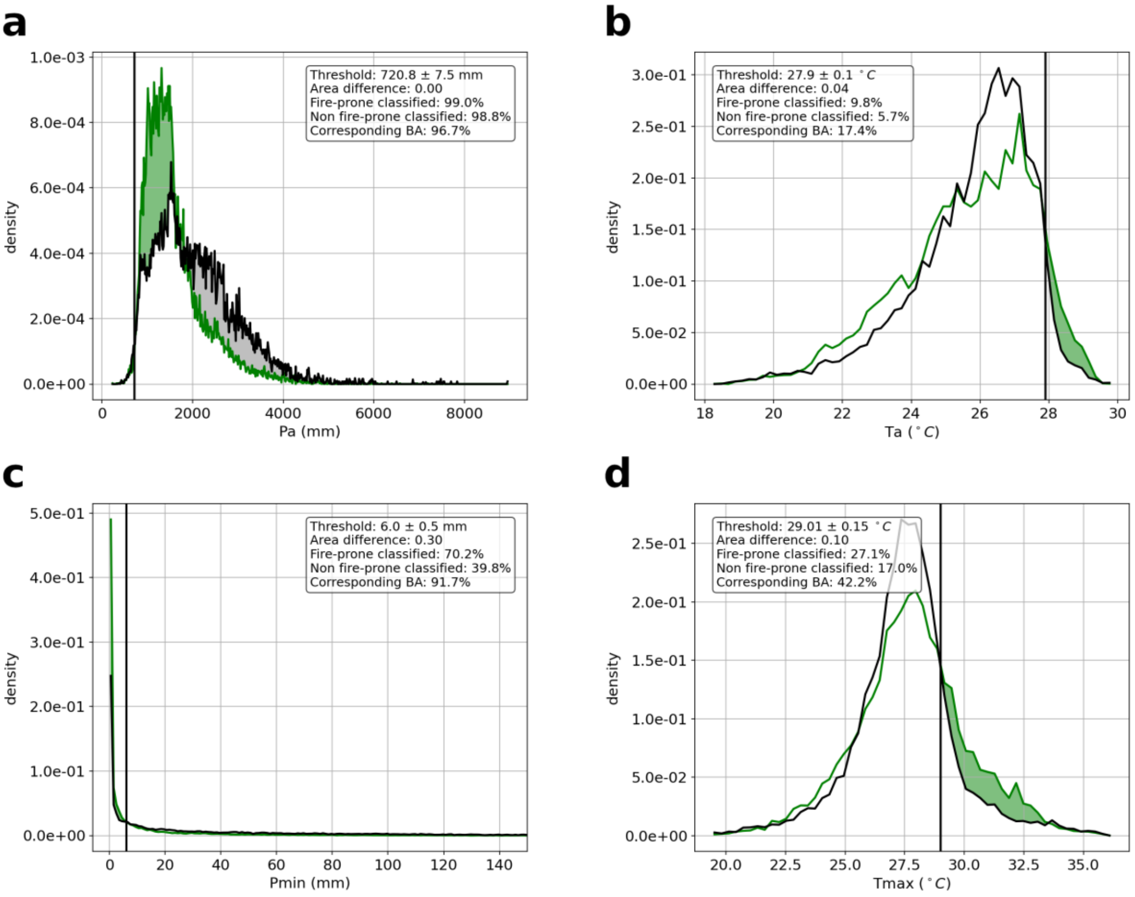

Supplementary Figure 5. Tropical statistical distributions for annual variables. Annual precipitation - Pa (**a**), annual mean temperature - Ta (**b**), precipitation of the driest month - Pmin (**c**) and temperature of the hottest month - Tmax (**d**). Green density functions represent values for cells and years with BA≥100 ha, and black density functions represent values for cells and years with BA<100 ha. The thresholds are indicated through black vertical lines. The text box shows the value of the threshold and its uncertainty, the value of the area difference between fire and non-fire points, the percentage of fire points meeting the threshold, the percentage of non-fire points meeting the threshold and the percentage of burned area associated with the fire points that meet the threshold. The area difference is proportional to the difference between the percentage of fire and non-fire points. The uncertainty in the obtained threshold is the size of the x-axis discretization used in the calculation.

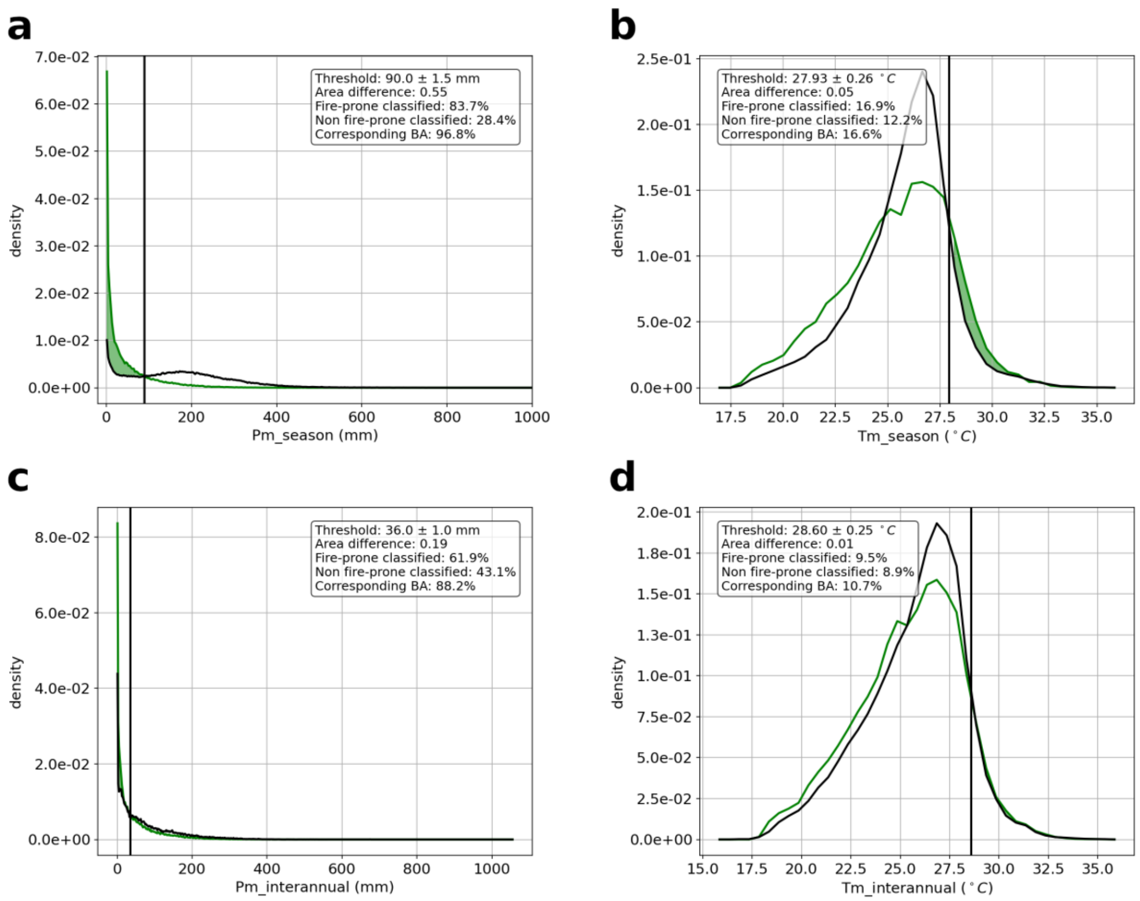

Supplementary Figure 6. Tropical statistical distributions for monthly variables. Monthly precipitation and temperature seasonal distributions (**a**, **b**) and interannual distributions (**c**, **d**). The thresholds are indicated through black vertical lines. In the seasonal distributions, green density functions represent the FS months of the BA≥100 ha years, and black density functions represent the months out of the FS. In the interannual distributions, green density functions represent the FS of the BA≥100 ha years, and black density functions represent the FS of the BA<100 ha years. The thresholds are indicated through black vertical lines. The text box shows the value of the threshold and its uncertainty, the value of the area difference between fire and non-fire points, the percentage of fire points meeting the threshold, the percentage of non-fire points meeting the threshold and the percentage of burned area associated with the fire points that meet the threshold. The area difference is proportional to the difference between the percentage of fire and non-fire points. The uncertainty in the obtained threshold is the size of the x-axis discretization used in the calculation.

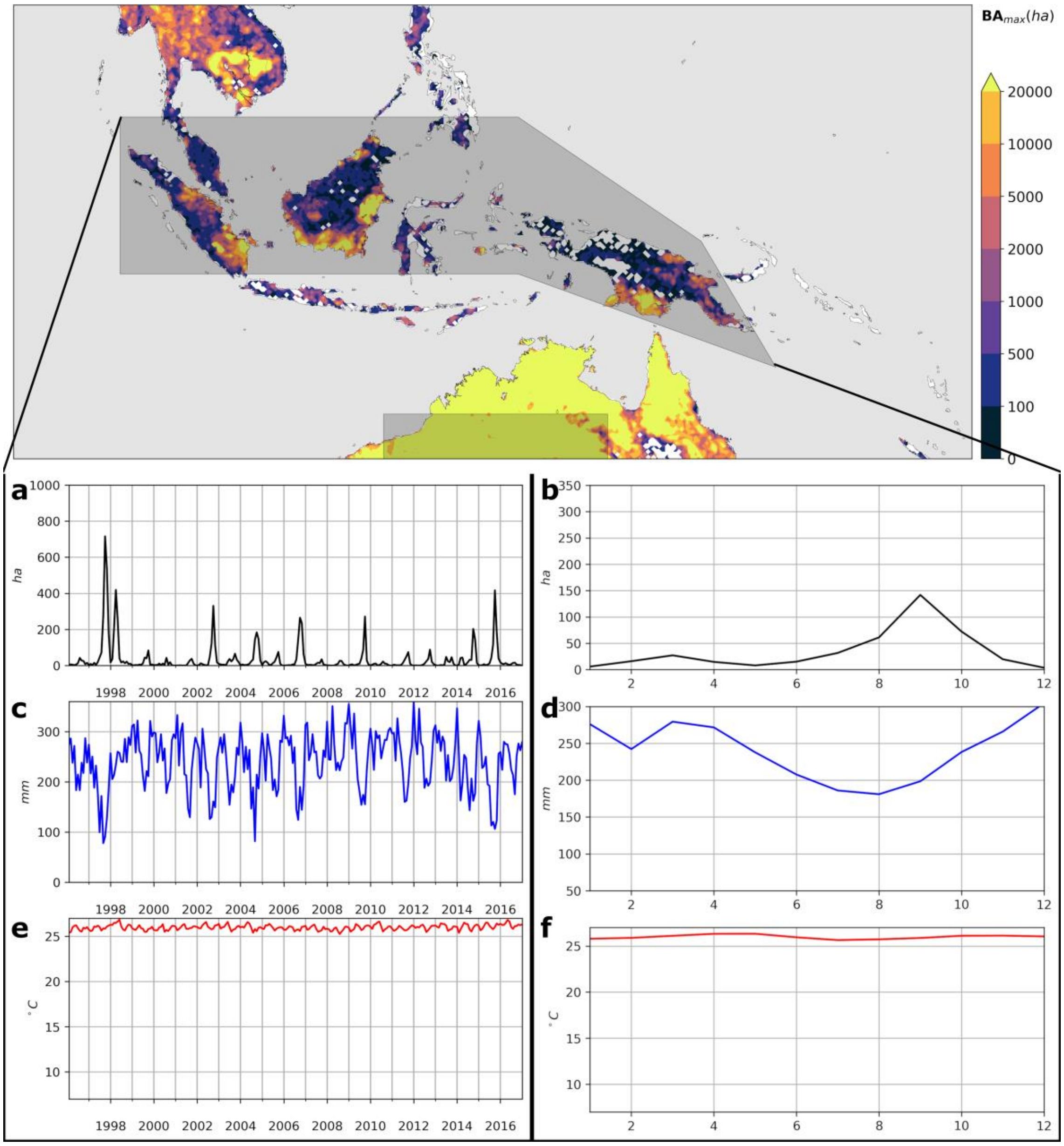

Supplementary Figure 7. Monthly values for South-eastern maritime Asia arid fire-prone regions. 1996-2016 time series of mean monthly values (**a**, **c**, **e**) and the mean annual cycles (**b**, **d**, **f**) of burned area (**a**, **b**, in black), precipitation (**c**, **d**, in blue) and 2m air temperature (**e**, **f** in red) for the Malay Archipelago fire-prone region (BA_{max}>0ha).

Arid fuel limited fire category

An arid fuel limited fire category (Ar-fl) is initially defined through a Pa (Supplementary Fig. 8a) and a Tm threshold (Supplementary Fig. 9b), with area differences of 0.44 and 0.23 respectively. Fires are observed in regions with an annual precipitation that is sufficient to enable the existence of a certain amount of vegetation, so fire in dry climates is more limited

by fuel amount than by fuel moisture[19,20]. The Pa arid threshold is applied to all other categories to analyse a possible future desertification due to climate change. High monthly temperatures are also related to fire activity. Supplementary Fig. 9b shows that most of the fire season months in arid climates have temperatures above 19.5°C. Despite monthly precipitation distributions not showing significant differences between fire-prone and non-fire-prone data, we also include a Pm threshold of 60 mm (corresponding to the 90th percentile of the fire points distribution) in order to detect a possible fire season shortening due to a future precipitation increase. Above this monthly precipitation value, the number of months belonging to a fire season anywhere with arid climate is almost negligible.

However, these three thresholds (Pa, Tm and Pm) are not enough to characterize the fire season in certain regions. We checked the monthly values of burned area, precipitation and temperature in different fire-prone arid regions (Supplementary Fig. 10, 11, 12 and 13) and found that the fire season is associated with a hot season in midlatitude arid areas where no clear wet period is observed, e.g. Kazakhstan and North America (Supplementary Fig. 10 and 11), but closer to the tropics where it is warm year-round, it can be also determined by the existence of a pronounced annual wet and dry season cycle, with fires occurring sometime during the dry season, in places just at the beginning (e.g. Sahel region, Supplementary Fig. 12) while in others, more toward the end of it (e.g. Botswana and Australia, Supplementary Fig. 12 and 13).

Supplementary Fig. 14 shows the distance in months between the month of maximum burned area and the wettest month (Supplementary Fig. 14a), and between the month of maximum burned area and the hottest month (Supplementary Fig. 14b). The values of the latter variables indicate that the maximum burned area in the cool arid regions (e.g. Kazakhstan and North America) is reached during the hottest months, so that the Tm threshold can be sufficient to define the fire season. However, in warmer regions with more tropical monsoonal climatic features, additional variables must be considered. Supplementary Fig. 15a compares the mean annual temperature (MAT) probability density function of areas where the month of maximum burned area precede the wettest month, i.e., it is at the end of the dry season (yellow distribution) vs. areas where the month of maximum burned area follows the wettest month, i.e., it is at the beginning of the dry season (orange distribution). According to this, we define three different arid fire-prone regions:

(a) MAT < 18.5°C. Due to the practical absence of precipitation seasonality (Supplementary Fig. 10 and 11), the fire season coincides with the hot season (Supplementary Fig. 15b).

(b) 18.5°C ≤ MAT < 27.5°C. The fire season occurs at the end of the dry months, just before the wet season, when the fuel moisture reaches the lowest values (Supplementary Fig. 15c). For this MAT interval we will constrain the fire season to the five months at the end of the dry season.

(c) MAT ≥ 27.5°C. The high temperatures rapidly dry the fuel, so the fire season occurs at the beginning of the dry season, right after the wet season (Supplementary Fig. 15d). Two more factors enhance the rapid fuel drying in the Sahel region, the low soil water retention[21] and the grass-dominated vegetation[22] in contrast with other arid regions covered mostly by shrubs or hummock grasses, as in Australia. The herbaceous vegetation desiccates early in the dry season[23], favouring fires, while the severe drought suppresses grass production at the end of the dry season, limiting fires[23]. In addition, fires in the early dry season are used as preventive burning strategies in order to hinder later destructive fires in Mali[24] and Senegal[25]. Therefore, for these MAT values we will constrain the fire season to the three months at the beginning of the dry season.

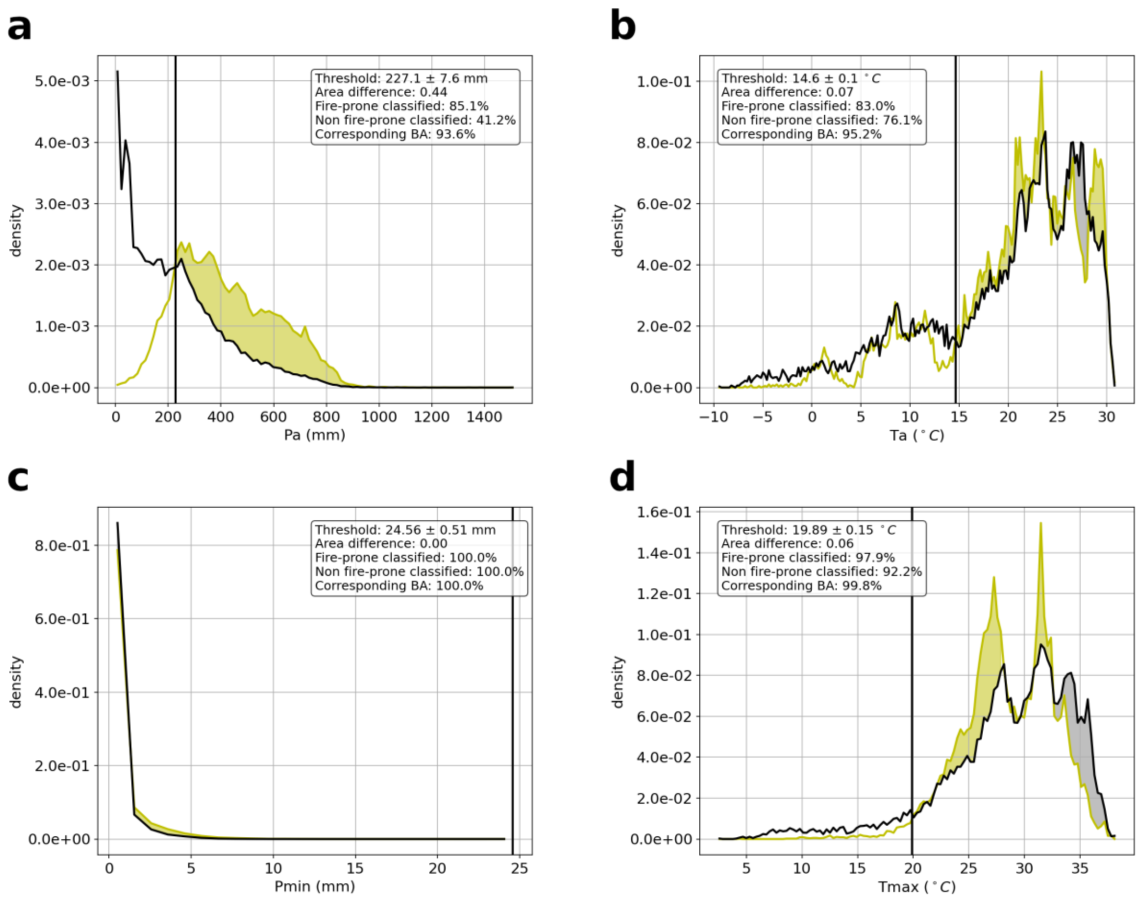

Supplementary Figure 8. Arid statistical distributions for annual variables. Annual precipitation - Pa (**a**), annual mean temperature - Ta (**b**), precipitation of the driest month - Pmin (**c**) and temperature of the hottest month - Tmax (**d**). Yellow density functions represent values for cells and years with BA≥100 ha, and black density functions represent values for cells and years with BA<100 ha. The thresholds are indicated through black vertical lines. The text box shows the value of the threshold and its uncertainty, the value of the area difference between fire and non-fire points, the percentage of fire points meeting the threshold, the percentage of non-fire points meeting the threshold and the percentage of burned area associated with the fire points that meet the threshold. The area difference is proportional to the difference between the percentage of fire and non-fire points. The uncertainty in the obtained threshold is the size of the x-axis discretization used in the calculation.

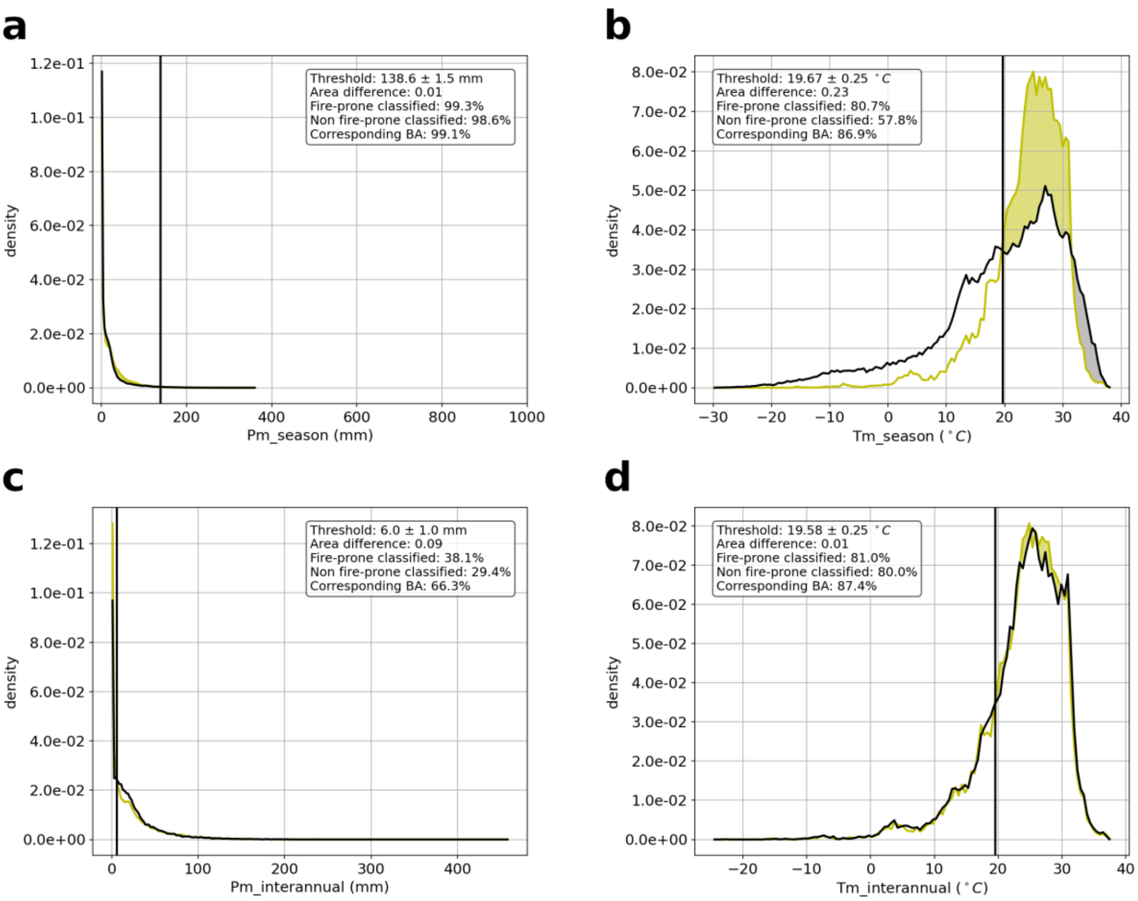

Supplementary Figure 9. Arid statistical distributions for monthly variables. Monthly precipitation and temperature seasonal distributions (**a**, **b**) and interannual distributions (**c**, **d**). The thresholds are indicated through black vertical lines. In the seasonal distributions, yellow density functions represent the FS months of the BA≥100 ha years, and black density functions represent the months out of the FS. In the interannual distributions, yellow density functions represent the FS of the BA≥100 ha years, and black density functions represent the FS of the BA<100 ha years. The thresholds are indicated through black vertical lines. The text box shows the value of the threshold and its uncertainty, the value of the area difference between fire and non-fire points, the percentage of fire points meeting the threshold, the percentage of non-fire points meeting the threshold and the percentage of burned area associated with the fire points that meet the threshold. The area difference is proportional to the difference between the percentage of fire and non-fire points. The uncertainty in the obtained threshold is the size of the x-axis discretization used in the calculation.

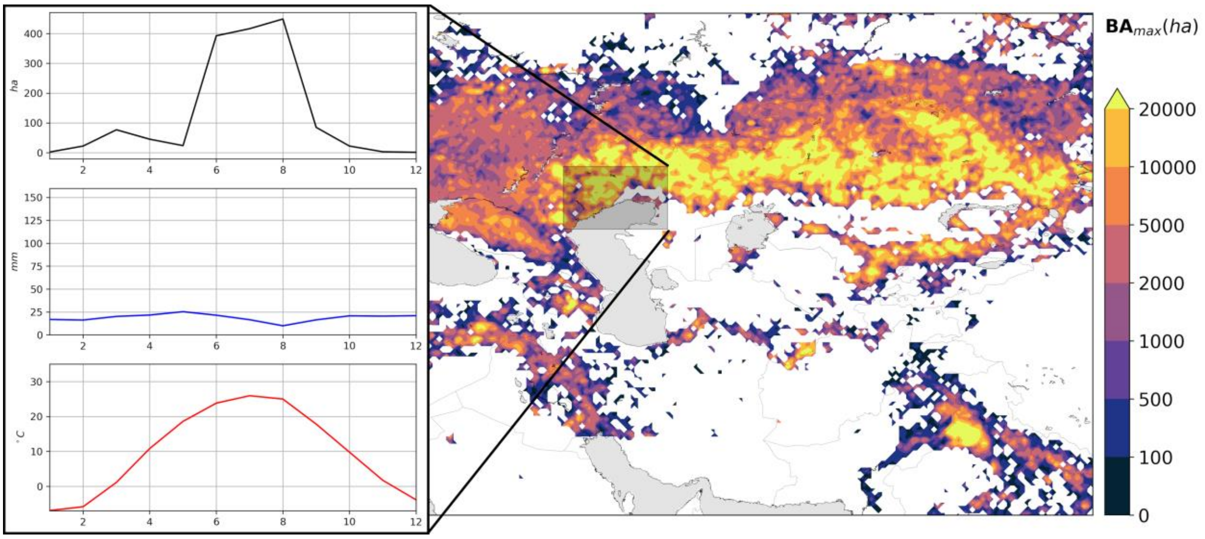

Supplementary Figure 10. Mean monthly values for Asian arid fire-prone regions (BA_{max}>0ha). Mean monthly burned area in black, precipitation in blue and 2m air temperature in red.

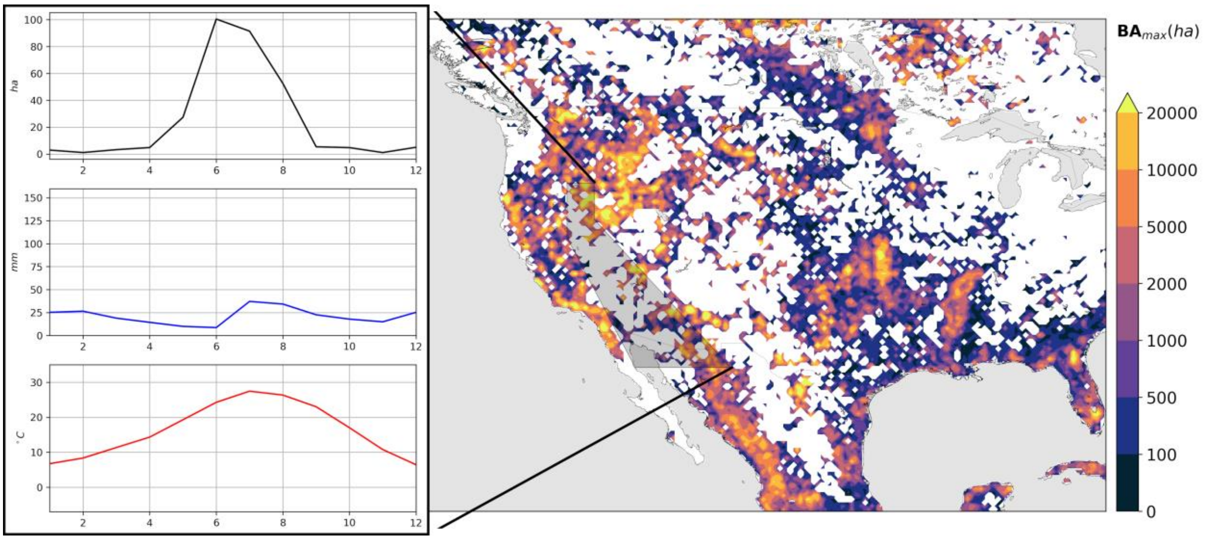

Supplementary Figure 11. Mean monthly values for North American arid fire-prone regions (BA_{max}>0ha). Mean monthly burned area in black, precipitation in blue and 2m air temperature in red.

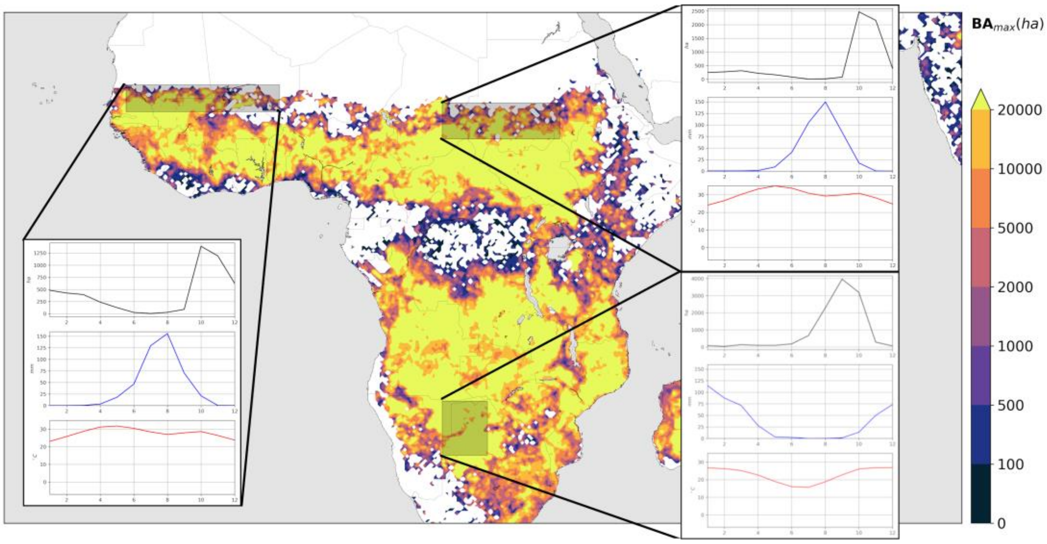

Supplementary Figure 12. Mean monthly values of African fire-prone arid regions (BA_{max}>0ha). Mean monthly burned area in black, mean monthly precipitation in blue and mean monthly 2m air temperature in red.

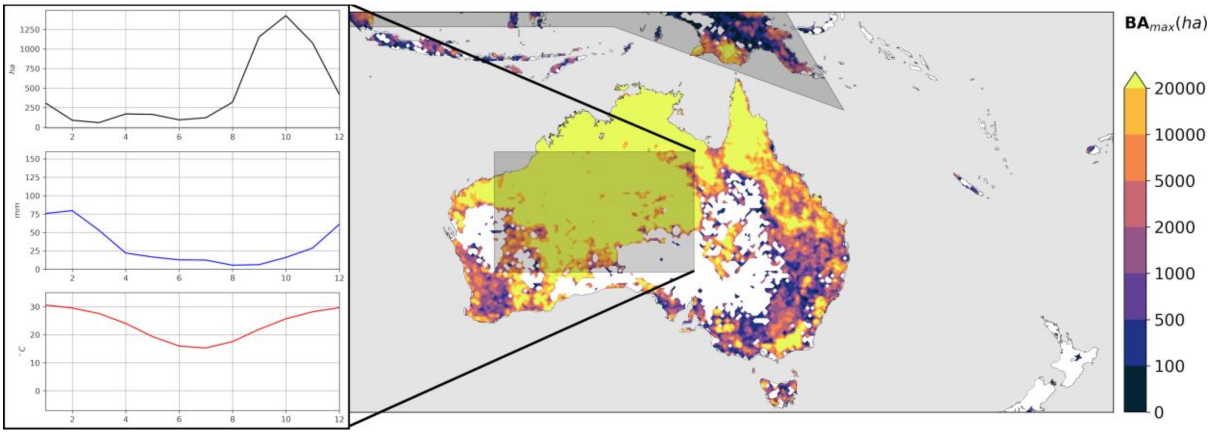

Supplementary Figure 13. Mean monthly values for Australian arid fire-prone regions (BA_{max}>0ha). Mean monthly burned area in black, mean monthly precipitation in blue and mean monthly 2m air temperature in red.

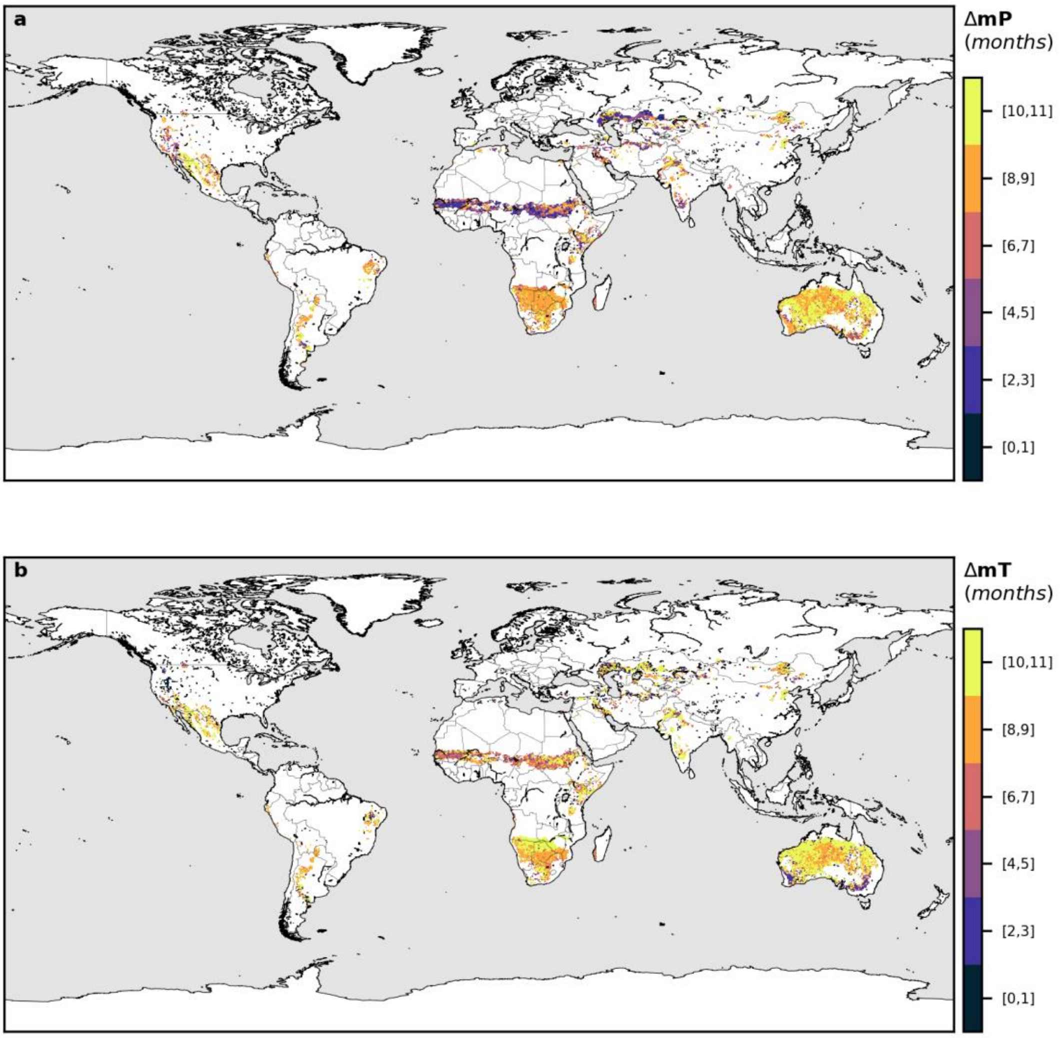

Supplementary Figure 14. Months from the month with highest burned area. a Time period in months between the month with most burned area and the wettest month (ΔmP). **b** Time period in months between the month with most burned area and the hottest month (ΔmT).

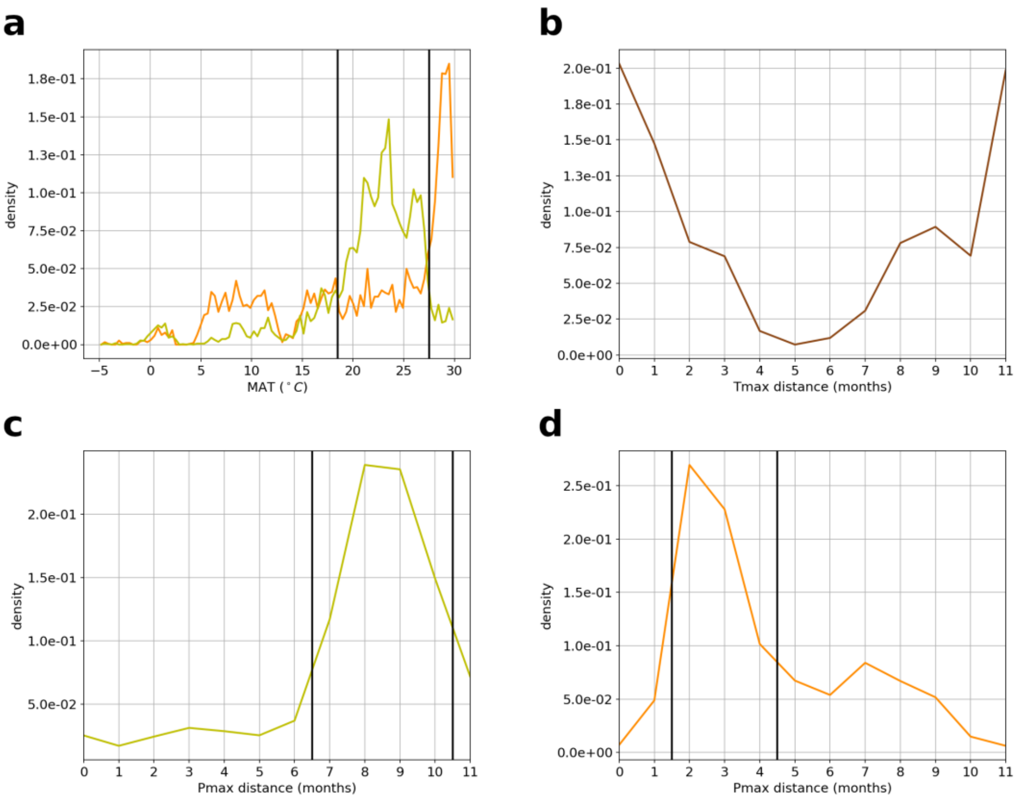

Supplementary Figure 15. Auxiliar statistical distributions for arid climates. a MAT probability density function of arid fire-prone points ($BA_{max} \geq 0$ ha) with $\Delta mP>6$, i.e. with more than 6 months in between the month with most burned area and the wettest month (yellow) and with $\Delta mP \leq 6$ values (orange). **b** For points with MAT < 18.5°C, probability density function of the distance in months between fire season months and the hottest month (brown). **c** For points with 18.5°C ≤ MAT < 27.5°C, probability density function of the distance in months between fire season months and the wettest month (yellow). **d** For points with MAT ≥ 27.5°C, probability density function of the distance in months between fire season months and the wettest month (orange).

Temperate dry and hot season fire category

The Temperate dry and hot season fire category (Te-dhs) is defined by setting a Pm and a Pmin threshold, indicating a precipitation seasonality dependence as in the Tropics. The fire season months are relatively dry; most of the Pm values are concentrated below a 42 mm threshold (Supplementary Fig. 17a), with a pronounced peak close to 0 mm, meaning a totally dry month. The Tm seasonal distribution (Supplementary Fig. 17b) shows also significant differences between fire season months and non-fire-prone months, indicating that cooler monthly temperature values below 12°C tend to hamper fire activity. Therefore, the fire season in temperate climates occurs during dry and hot conditions. As we see in Supplementary Fig. 16 and 17, Pm, Tm and Pmin are the three variables that show the highest area differences (0.34 and 0.33 and 0.29, respectively), with the fire points classified representing more than the 88% of the burned area data.

Unlike in ever warm tropical climates, where fire risk is largely determined by the lack of precipitation in the dry season, in midlatitude temperate climates with temperature seasonality, fires occur when dry conditions coincide with the warm season[26-31], as cold temperatures diminish fire risk. Thus, the use of simultaneous monthly precipitation and temperature thresholds are needed. The annual Ta and Pa statistical distributions (Supplementary Fig. 16a,b) do not show any important distinction in temperate fire-prone regions. With regard to interannual variability, the years of high burned area show slightly drier fire seasons (Supplementary Fig. 17c), but the differences at this broad spatial scale of analysis are so small that we choose not to modify the Pm threshold.

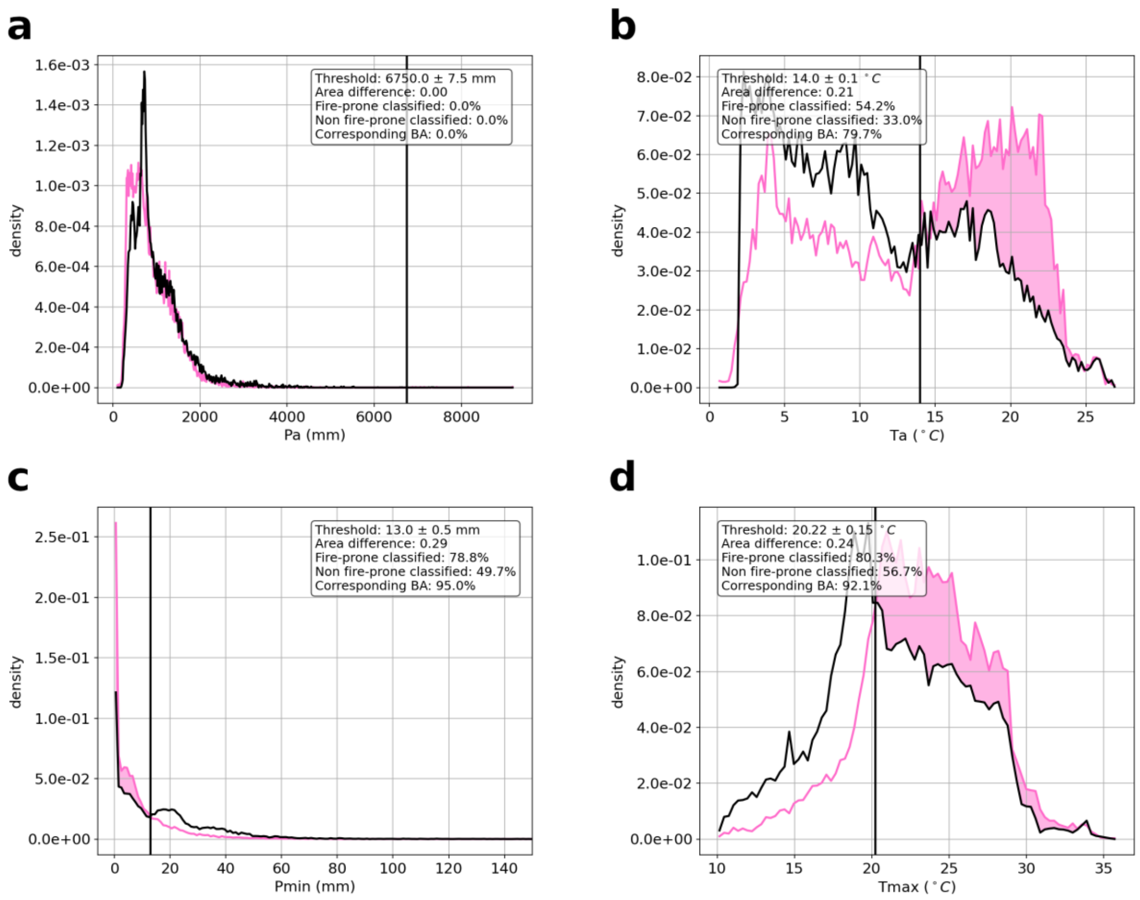

Supplementary Figure 16. Temperate statistical distributions for annual variables. Annual precipitation - Pa (**a**), annual mean temperature - Ta (**b**), precipitation of the driest month - Pmin (**c**) and temperature of the hottest month - Tmax (**d**). Pink density functions represent values for cells and years with BA≥100 ha, and black density functions represent values for cells and years with BA<100 ha. The thresholds are indicated through black vertical lines. The text box shows the value of the threshold and its uncertainty, the value of the area difference between fire and non-fire points, the percentage of fire points meeting the threshold, the percentage of non-fire points meeting the threshold and the percentage of burned area associated with the fire points that meet the threshold. The area difference is proportional to the difference between the percentage of fire and non-fire points. The uncertainty in the obtained threshold is the size of the x-axis discretization used in the calculation.

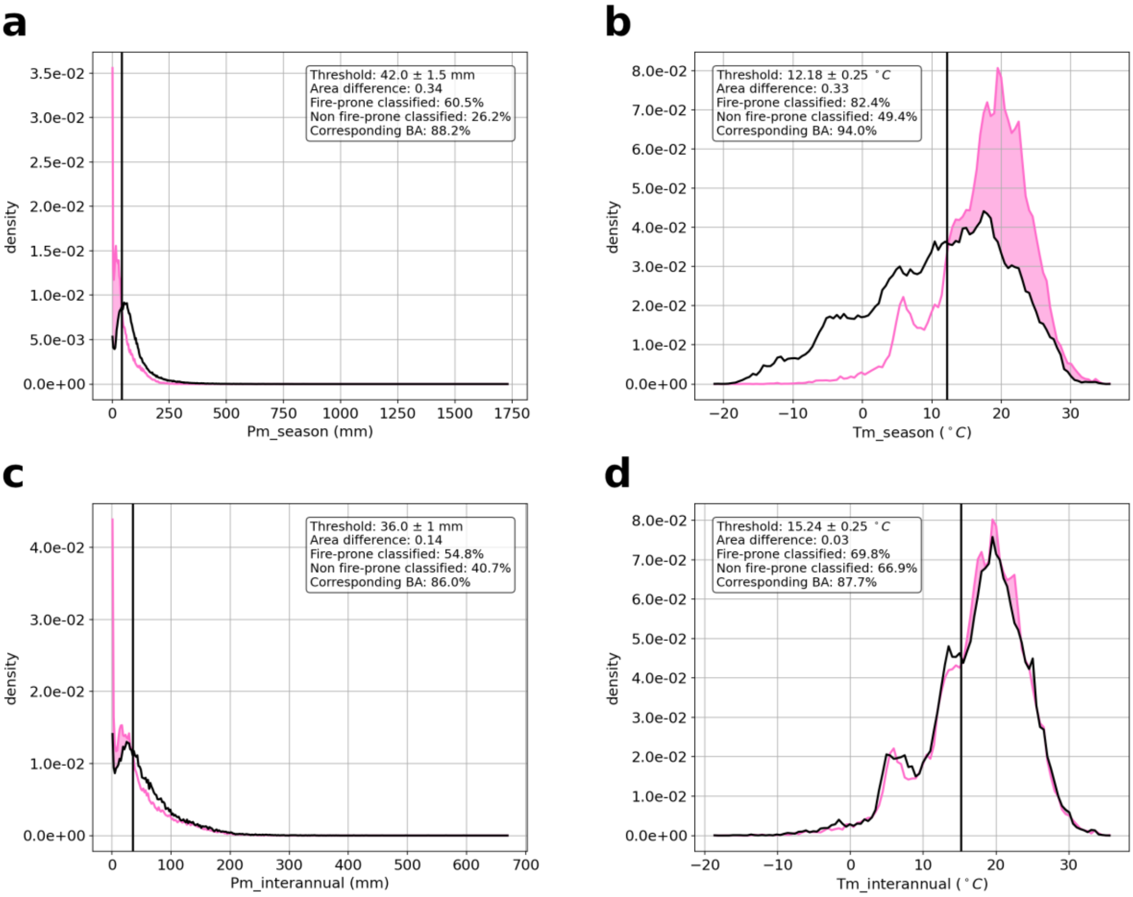

Supplementary Figure 17. Temperate statistical distributions for monthly variables. Monthly precipitation and temperature seasonal distributions (**a**, **b**) and interannual distributions (**c**, **d**). The thresholds are indicated through black vertical lines. In the seasonal distributions, pink density functions represent the FS months of the BA≥100 ha years, and black density functions represent the months out of the FS. In the interannual distributions, pink density functions represent the FS of the BA≥100 ha years, and black density functions represent the FS of the BA<100 ha years. The thresholds are indicated through black vertical lines. The text box shows the value of the threshold and its uncertainty, the value of the area difference between fire and non-fire points, the percentage of fire points meeting the threshold, the percentage of non-fire points meeting the threshold and the percentage of burned area associated with the fire points that meet the threshold. The area difference is proportional to the difference between the percentage of fire and non-fire points. The uncertainty in the obtained threshold is the size of the x-axis discretization used in the calculation.

Boreal hot season fire category

The Boreal hot season fire category (Bo-hs) is mainly defined through a Tmax and a Tm threshold, given that the fire season is characterized by the warmest conditions along the year. Supplementary Fig. 19b clearly shows how most fire season months have monthly temperatures above 7°C. In a similar way as we did for the precipitation thresholds in Tropical climates, we combine this monthly threshold with a maximum monthly temperature threshold of 15°C (Supplementary Fig. 18d). This indicates that boreal regions with a warm season with more moderate monthly temperatures ranging between 7°C and 15°C are not classified as fire prone.

It is typical of boreal climates to present higher precipitation in warm months, as the very cold air masses in winter contain little moisture and precipitation amounts are commonly small, especially in regions away from the coast. This is the reason why the monthly precipitation seasonal statistical distribution (Supplementary Fig. 18a) does not show a drier fire season than the rest of the year under Boreal climates. However, Supplementary Fig. 18c clearly indicates that the fire season in the particular years with burned area is noticeable drier than in years with no fires, which means that boreal fires, in addition to the need of warm conditions to occur, easily achieved almost every year, are enhanced when the warm season coincides with a dry period. This explains why boreal fires have the lowest annual frequency among all main fire classes (Fig. 2b of the main text), and suggests the need of an additional threshold for monthly precipitation to better characterize the climate setting in which they develop. Because the obtained threshold from the distributions of Supplementary Fig. 18c is too restrictive (with only 49.7% of the fire points meeting the condition) we use the 75th percentile of the fire point distribution, corresponding to 67 mm, which still includes a sizeable amount of non-fire points, but discards many, nevertheless.

Due to the practical absence of polar (E) fires, as cold temperatures are registered year around and vegetation consists of tundra, we excluded this general climate class from our analysis.

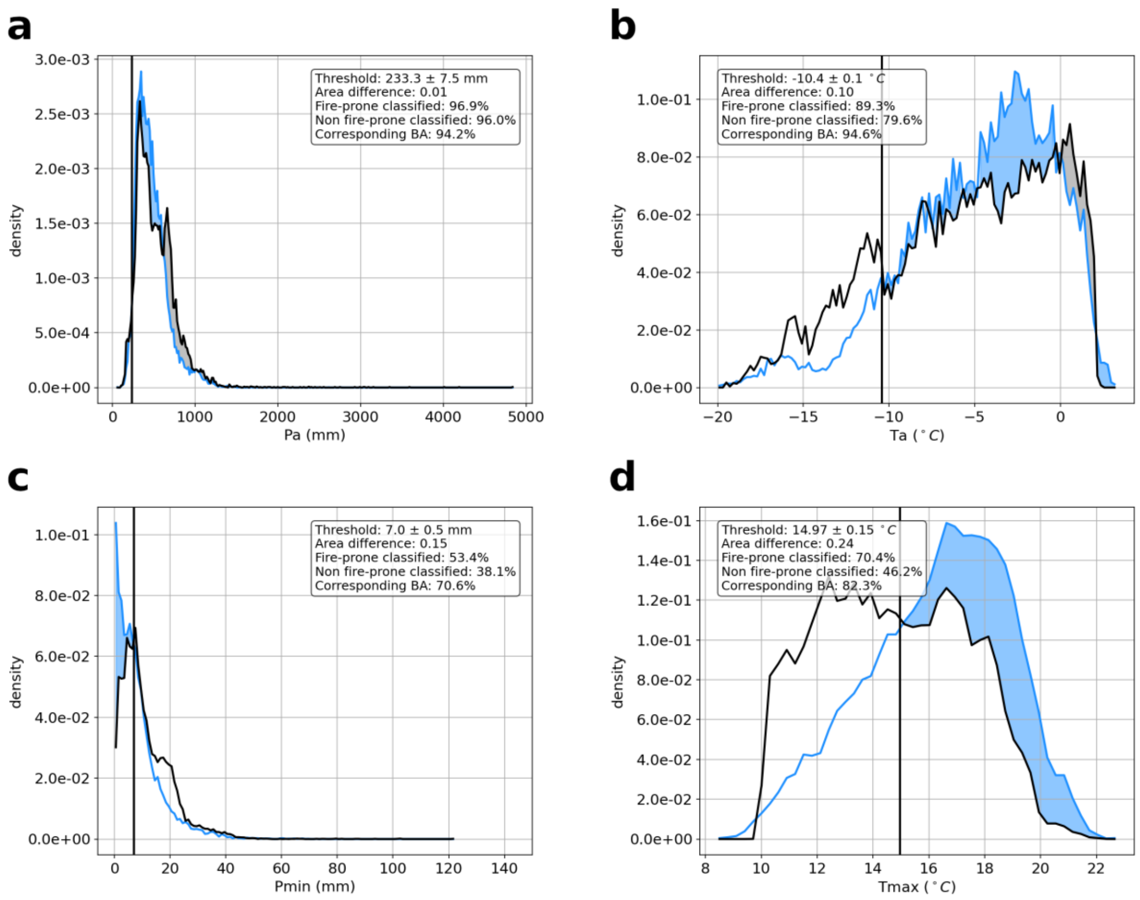

Supplementary Figure 18. Boreal statistical distributions for annual variables. Annual precipitation - Pa (**a**), annual mean temperature - Ta (**b**), precipitation of the driest month - Pmin (**c**) and temperature of the hottest month - Tmax (**d**). Blue density functions represent values for cells and years with BA≥100 ha, and black density functions represent values for cells and years with BA<100 ha. The thresholds are indicated through black vertical lines. The text box shows the value of the threshold and its uncertainty, the value of the area difference between fire and non-fire points, the percentage of fire points meeting the threshold, the percentage of non-fire points meeting the threshold and the percentage of burned area associated with the fire points that meet the threshold. The area difference is proportional to the difference between the percentage of fire and non-fire points. The uncertainty in the obtained threshold is the size of the x-axis discretization used in the calculation.

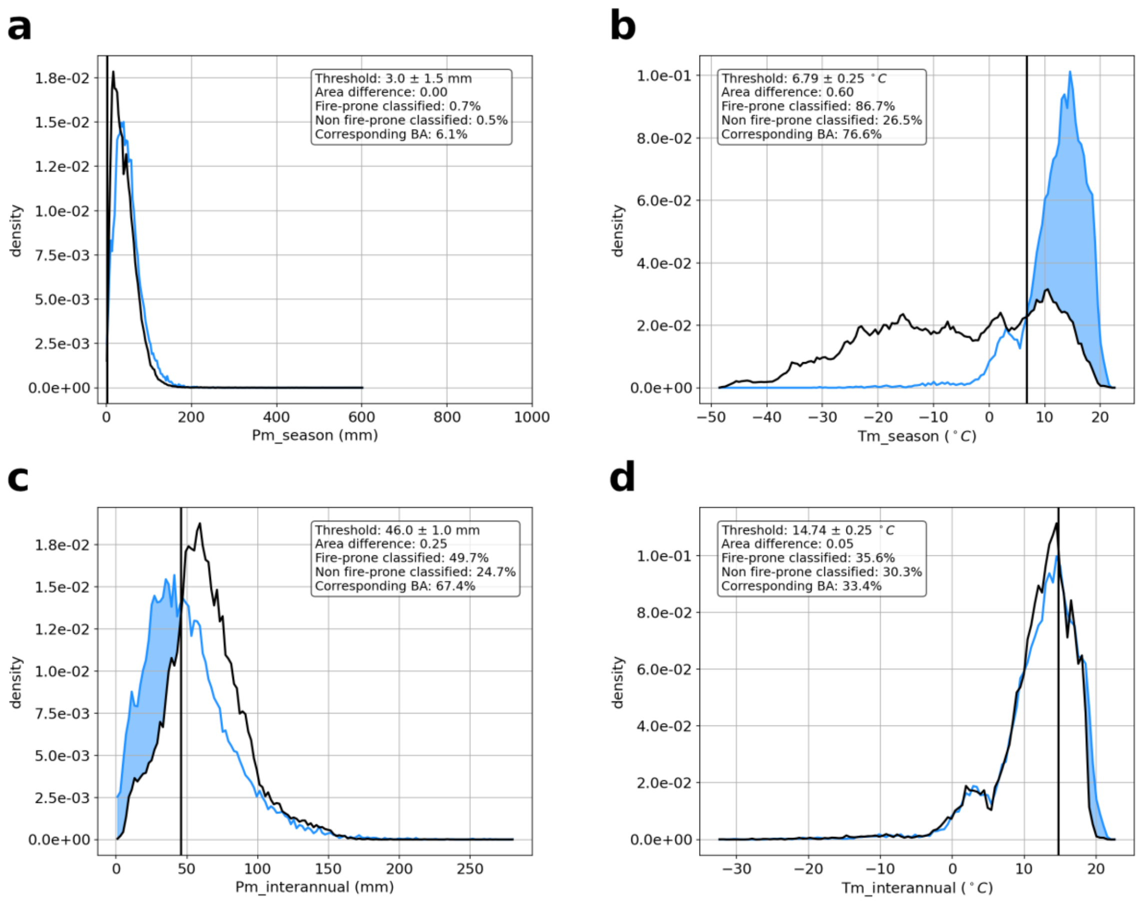

Supplementary Figure 19. Boreal statistical distributions of monthly variables. Monthly precipitation and temperature seasonal distributions (**a**, **b**) and interannual distributions (**c**, **d**). The thresholds are indicated through black vertical lines. In the seasonal distributions, blue density functions represent the FS months of the BA≥100 ha years, and black density functions represent the months out of the FS. In the interannual distributions, blue density functions represent the FS of the BA≥100 ha years, and black density functions represent the FS of the BA<100 ha years. The thresholds are indicated through black vertical lines. The text box shows the value of the threshold and its uncertainty, the value of the area difference between fire and non-fire points, the percentage of fire points meeting the threshold, the percentage of non-fire points meeting the threshold and the percentage of burned area associated with the fire points that meet the threshold. The area difference is proportional to the difference between the percentage of fire and non-fire points. The uncertainty in the obtained threshold is the size of the x-axis discretization used in the calculation.

Fire-climate classification reliability

We note that some fire-impacted areas have not been classified (in grey in Fig. 2a of the main text). These areas represent the 8.78% of the total fire-impacted global land area (points with BA_{max}>0ha), as shown in Supplementary Fig. 20a. Notwithstanding, they correspond for the most part to areas with relatively low fire incidence, as combined they only account for less than 1% of the global mean annual burned area (Supplementary Fig. 20a). The climate with the lowest amount of burned area observed at classified grid cells is the Boreal climate (89.08%, Supplementary Fig. 20a).

On the contrary, some regions with BA_{max}=0ha are classified as fire-prone, represented as black points in Fig. 2a of the main text and Supplementary Fig. 20b. Even though the percentage of these black points could seem very large (Supplementary Fig. 20b), this is mostly due to the discontinuous nature of the observed burned area data (Fig. 1 of the main text). In addition, a large percentage of these missclassified regions with observed BA_{max}=0ha (49.03%) are classified as infrequently fire-prone (Tr-ds-i, Ar-fl-i, Te-ds-i and Bo-hs-i). Few broad regions are represented in black in Fig. 2a of the main text. Most of them correspond to boundaries between arid fire-prone areas (Ar-fl) and deserts (e.g. Afghanistan, Ethiopia, Niger or Australia), which are difficult to define based solely on annual precipitation. In the Scandinavian Peninsula the burned area data do not reflect high fire activity. However, our classification identifies a fire-prone region from Northwest Russia towards the North of Sweden. The mismatch between our results and the burned area data in this case could be due to the effective fire suppression system in Nordic countries like Finland[83]. In terms of the global land area, 71.91% of area is well classified (classified BA>0ha points and non-classified BA=0ha points, Supplementary Fig. 20b).

Although there may be coincidences between our climate classification and fire regimes, e.g. infrequent fires in Boreal regions (Bo-hs) or frequent fires in the Tropics (Tr-ds)[2], our classification is not related to fire regimes because these are strongly dependent on other factors such as vegetation traits[33] or human action[34].

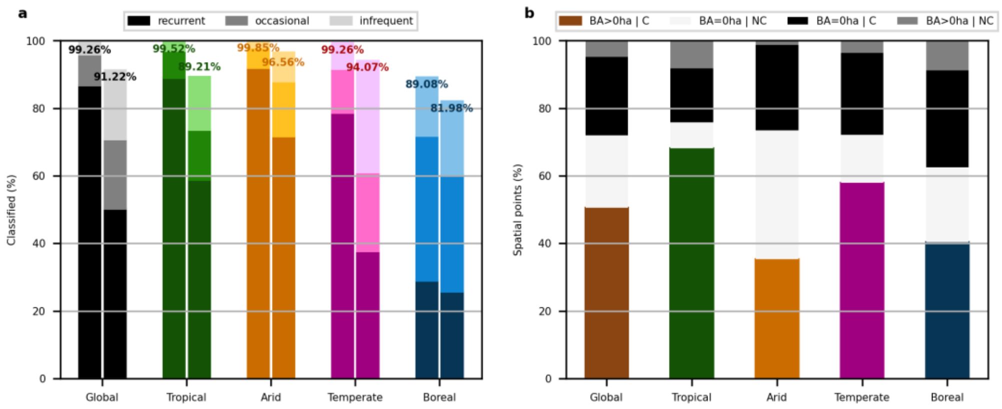

Supplementary Figure 20. Validation data of the fire-climate classification. a Percentage of global mean annual burned area observed at cells classified as fire-prone (left bars) and percentage of grid cells with non-zero burned area classified as fire-prone (right bars). **b** Percentage of: classified (color) and non-classified (black) points with non-zero observed burned area and classified (grey) and non-classified (white) with zero observed burned area (quantification of Fig. 2a of the main text).

Future changes in precipitation and temperature

To understand the factors behind the future changes in the spatial expansion of the fire-prone regions and in the length of the potential fire season, we examine the precipitation and temperature differences between future and present data. The global future change projections for mean annual precipitation (MAP) and mean annual temperature (MAT) are shown in Supplementary Fig. 21. In Supplementary Fig. 22 and 23 we represent the future changes of mean seasonal precipitation and temperature, respectively.

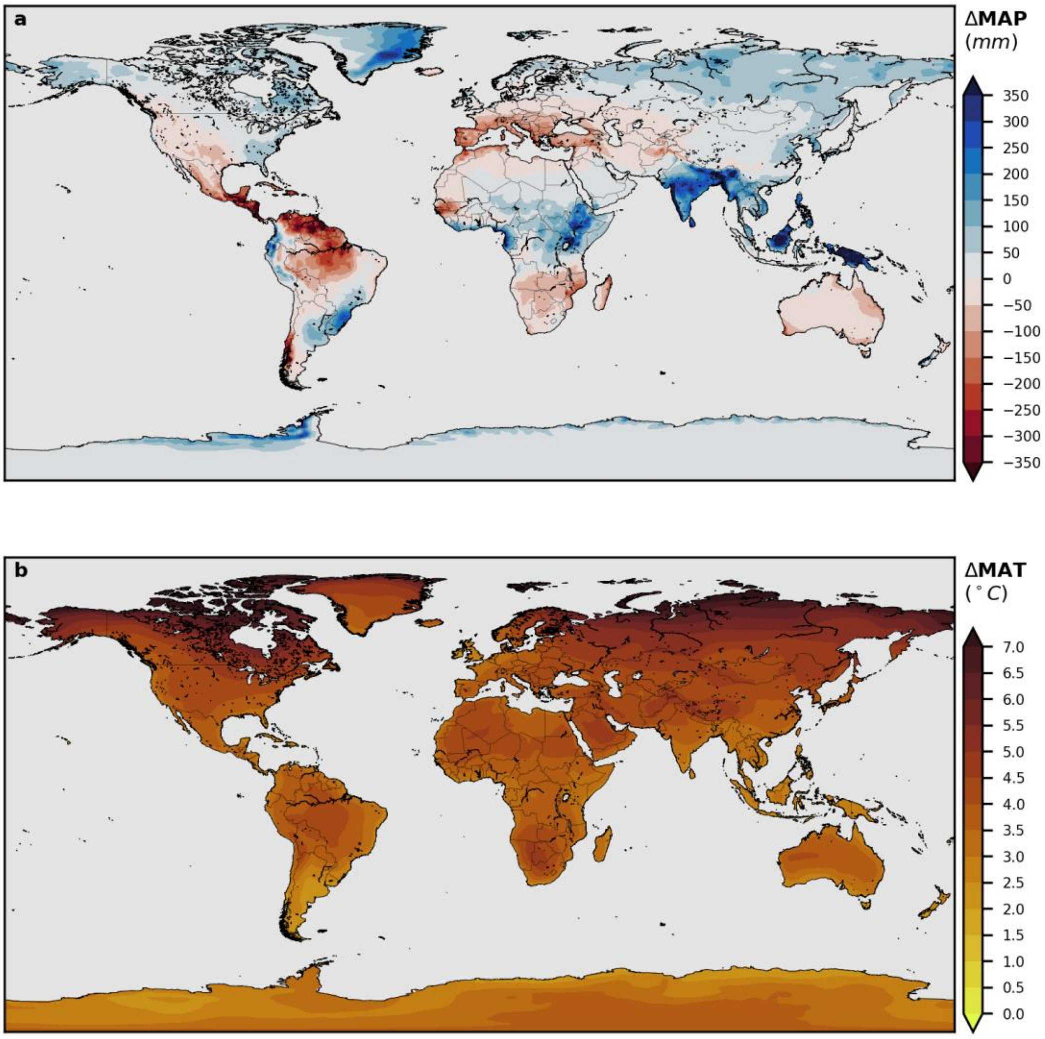

Supplementary Figure 21. Future changes in annual mean precipitation and temperature. a Difference between 2070-2099 MAP and 1996-2016 MAP (ΔMAP). **b** Difference between 2070-2099 MAT and 1996-2016 MAT (ΔMAT).

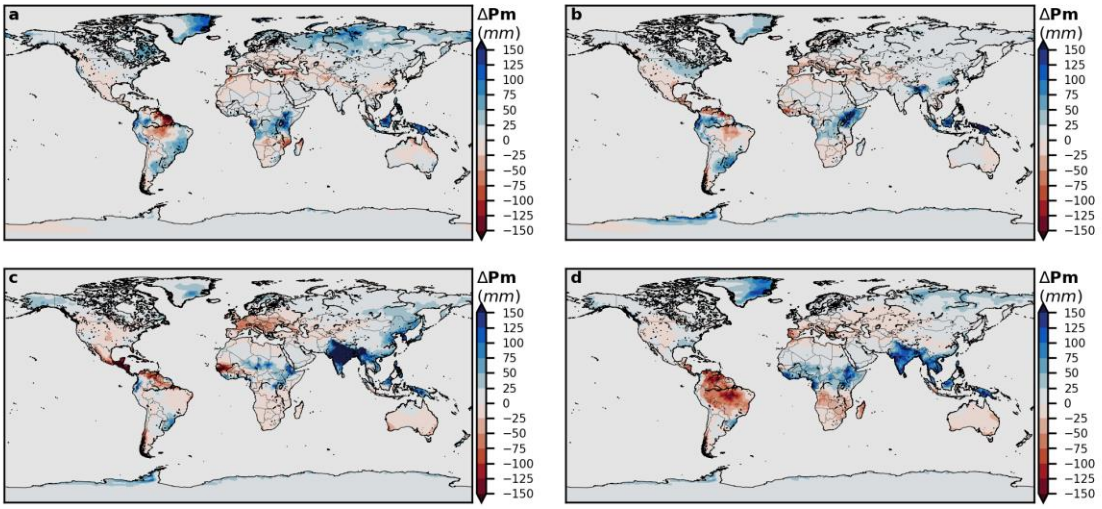

Supplementary Figure 22. Future changes in precipitation by season. Difference between 2070-2099 and 1996-2016 mean monthly precipitation for the DJF season (**a**), MAM season (**b**), JJA season (**c**) and SON season (**d**).

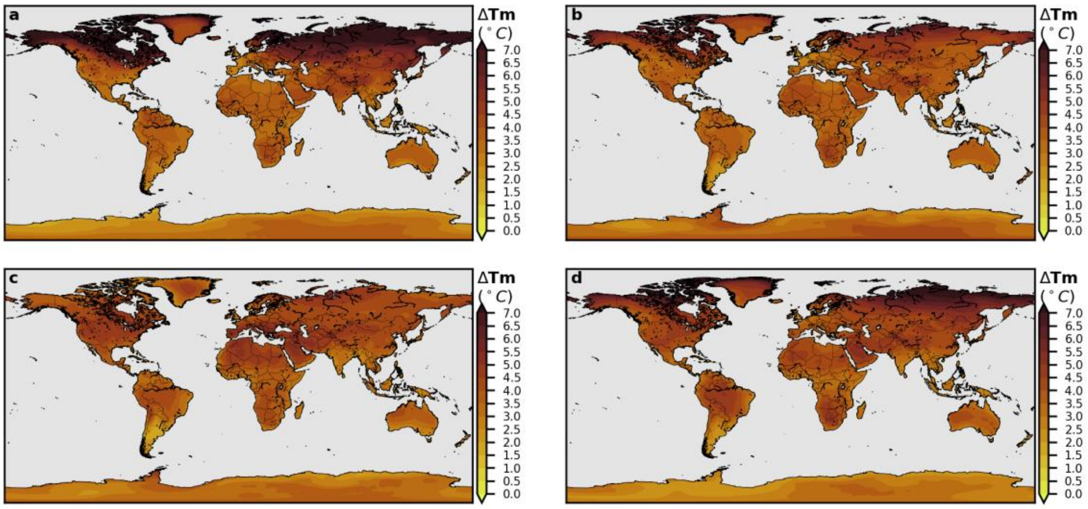

Supplementary Figure 23. Future changes in temperature by season. Difference between 2070-2099 and 1996-2016 mean monthly temperature for the DJF season (**a**), MAM season (**b**), JJA season (**c**) and SON season (**d**).

REFERENCES

1. Abatzoglou, J. T., Williams, A. P., Boschetti, L., Zubkova, M. & Kolden, C. A. Global patterns of interannual climate–fire relationships. *Glob. Chang. Biol.* **24**, 5164-5175 (2018).
2. Archibald, S., Lehmann, C. E., Gómez-Dans, J. L. & Bradstock, R. A. Defining pyromes and global syndromes of fire regimes. *Proc. Natl Acad. Sci. USA* **110,** 6442-6447 (2013).
3. Yue, C. et. al. Modelling the role of fires in the terrestrial carbon balance by incorporating SPITFIRE into the global vegetation model ORCHIDEE-Part 1: Simulating historical global burned area and fire regimes. *Geosci. Model Dev.* **7**, 2747–2767 (2014).
4. Giglio, L., Randerson, J. T. & Van der Werf, G. R. Analysis of daily, monthly, and annual burned area using the fourth-generation global fire emissions database (GFED4). *J. Geophys. Res. Biogeosc.* **118**, 317-328 (2013).
5. Köppen, W. *Das geographische System der Klimate,* 1-44 (Gebrüder Borntraeger: Berlin, Germany, 1936).
6. Beck, H. E. *et al.* Present and future Köppen-Geiger climate classification maps at 1-km resolution. *Sci. Data* **5**, 180214 (2018).
7. Peel, M. C., Finlayson, B. L. & McMahon, T. A. Updated world map of the Köppen-Geiger climate classification. *Hydrology and Earth System Sciences* **11,** 1633-1644 (2007).
8. Wilcock, A. A. Köppen after fifty years. *Annals of the Association of American Geographers* **58,** 12-28 (1968).
9. Russel, R. J. Dry climates of the United States: I. Climatic map. *University of California, Publications in Geography* **5,** 1-41 (1931).
10. Kottek, M., Grieser, J., Beck, C., Rudolf, B., & Rubel, F. World map of the Köppen-Geiger climate classification updated. *Meteorologische Zeitschrift* **15,** 259-263 (2006).
11. Whittaker, R. H. *Communities and Ecosystems.* 2nd ed. 167 (MacMillan, New York, 1975).
12. Woodward, F. I., Lomas, M. R., & Kelly, C. K. Global climate and the distribution of plant biomes. *Philosophical Transactions of the Royal Society of London. Series B: Biological Sciences*, **359,** 1465-1476 (2004).
13. Cucchi, M. *et al.* WFDE5: bias adjusted ERA5 reanalysis data for impact studies. *Earth Syst. Sci. Data* **12**, 2097–2120 (2020).

14. Cahoon, D. R., Stocks, B. J., Levine, J. S., Cofer, W. R. & O'Neill, K. P. Seasonal distribution of African savanna fires. *Nature* **359**, 812-815 (1992).
15. Archibald, S., Roy, D. P., van Wilgen, B. W., & Scholes, R. J. What limits fire? An examination of drivers of burnt area in Southern Africa. *Glob. Change Biol.* **15**, 613-630 (2009).
16. Van Der Werf, G. R., Randerson, J. T., Collatz, G. J., & Giglio, L. Carbon emissions from fires in tropical and subtropical ecosystems. *Glob. Change Biol.* **9**, 547-562 (2003).
17. Van Der Werf, G. R. et al. Continental-scale partitioning of fire emissions during the 1997 to 2001 El Nino/La Nina period. *Science* **303**, 73-76 (2004).
18. Chen, Y. et al. A pan-tropical cascade of fire driven by El Niño/Southern Oscillation. *Nat. Clim. Change* **7**, 906-911 (2017).
19. Bradstock, R. A. A biogeographic model of fire regimes in Australia: current and future implications. *Glob. Ecol. Biogeogr.* **19**, 145-158 (2010).
20. Pausas, J. G., & Ribeiro, E. The global fire–productivity relationship. *Glob. Ecol. Biogeogr.* **22**, 728-736 (2013).
21. Le Page, Y., Oom, D., Silva, J. M., Jönsson, P., & Pereira, J. M. Seasonality of vegetation fires as modified by human action: Observing the deviation from eco-climatic fire regimes. *Glob. Ecol. Biogeogr.* **19**, 575-588 (2010).
22. Andela, N. *et al.* Biomass burning fuel consumption dynamics in the tropics and subtropics assessed from satellite. *Biogeosci. Discuss.* **13**, 3717-3734 (2016).
23. Kull, C.A. & Laris, P. Fire ecology and fire politics in Mali and Madagascar. In: *Tropical Fire Ecology: Climate Change, Land Use, and Ecosystem Dynamics.* Ed. Cochrane, M., pp. 171-226 (Springer-Praxis, Heidelberg, 2009).
24. Laris, P. Burning the seasonal mosaic: preventative burning strategies in the wooded savanna of southern Mali. *Hum. Ecol.* **30**, 155-186 (2002).
25. Mbow, C., Nielsen, T. T., & Rasmussen, K. Savanna fires in east-central Senegal: distribution patterns, resource management and perceptions. *Hum. Ecol.* **28**, 561-583 (2000).
26. Littell, J. S., McKenzie, D., Peterson, D. L. & Westerling, A. L. Climate and wildfire area burned in western US ecoprovinces, 1916-2003. *Ecol. Appl.* **19**, 1003-1021 (2009).
27. Marlon, J. R. *et al.* Long-term perspective on wildfires in the western USA. *Proc. Natl Acad. Sci. USA* **109**, E535-E543 (2012).

28. Parisien, M. A., & Moritz, M. A. Environmental controls on the distribution of wildfire at multiple spatial scales. *Ecol. Monogr.* **79**, 127-154 (2009).

29. Seager, R. *et al.* Climatology, variability, and trends in the US vapor pressure deficit, an important fire-related meteorological quantity. *J. Appl. Meteor. Climatol.* **54**, 1121-1141 (2015).

30. Sousa, P. M., Trigo, R. M., Pereira, M. G., Bedia, J., & Gutiérrez, J. M. Different approaches to model future burnt area in the Iberian Peninsula. *Agric. For. Meteorol.* **202**, 11-25 (2015).

31. Turco, M. *et al.* On the key role of droughts in the dynamics of summer fires in Mediterranean Europe. *Sci. Rep.* **7**, 1-10 (2017).

32. Kilpeläinen, A., Kellomäki, S., Strandman, H., & Venäläinen, A. Climate change impacts on forest fire potential in boreal conditions in Finland. *Clim. Change* **103**, 383-398 (2010).

33. Archibald, S. et al. Biological and geophysical feedbacks with fire in the Earth system. *Environ. Res. Lett.* **13**, 033003 (2018).

34. Bowman, D. M. et al. The human dimension of fire regimes on Earth. *J. Biogeog.* **38**, 2223-2236 (2011).
